# Acute inflammatory response in the subcutaneous versus periprosthethic space after incisional hernia repair: an original article

**DOI:** 10.1186/1471-2482-14-91

**Published:** 2014-11-15

**Authors:** Rosalia Patti, Anna Maria Caruso, Paolo Aiello, Giuseppe Livio Angelo, Salvatore Buscemi, Gaetano Di Vita

**Affiliations:** Department of Surgical, Oncological and Stomatological Science, General Surgery Unit, University of Palermo, Via Liborio Giuffrè n 5, 90127 Palermo, Italy

**Keywords:** Cytokines, Drain fluid, Hernia repair, Inflammatory response, Prosthesis

## Abstract

**Background:**

The acute inflammatory response following mesh implantation has been often evaluated in vitro and in animal models. The aim of this study was to evaluate the acute inflammatory response near the prosthesis in human by analysing some inflammatory indicators.

**Methods:**

We used a cohort of twelve male patients affected by midline incisional hernia, who were admitted for surgical mesh repair. A suction drain was placed between the mesh and rectal muscles whereas, the other one was placed between the subcutaneous tissue and the oblique external sheath. The acute inflammatory response was analyzed by measuring the production of interleukin [IL]-1, IL-10, IL-1ra, C-Reactive Protein (CRP), total proteins, albumin and pH in the drain fluids.

**Results:**

The dynamics of CRP and ILs production resulted similar in both drainages. Comparing drain over mesh and subcutaneous drain at all times, IL-1 and CRP values always resulted significantly higher in the first one, whereas IL-1ra and IL-10 values were significantly higher in the last one. Total protein and albumin were similar in both drains at all time; only in the drain over mesh fluid, pH values resulted significantly reduced in the fourth post-operative day.

**Conclusions:**

Our data showed that an acute inflammatory reaction is present in both sites examined. However, it was significantly higher in the space after mesh implantation.

## Background

The use of a prostheses in hernia surgery of the abdominal wall has significantly reduced the incidence of recurrence. Therefore, some complications, such as seroma formation, chronic pain, feeling of foreign body, wound infection, abdominal wall stiffness and nerve entrapment complaints occur more frequently after mesh repair. They seem to be related to a higher inflammatory response to the prosthetic material [1].

The implantation of surgical biomaterials challenges the onset of a local inflammatory response syndrome, which is a non-systemic reaction to sterile infection (e.g., trauma, necrosis), microbiological infection, or a combination of both [2]. Mesh induced inflammation is linked to the foreign body granulation with alteration in the collagen composition [3]. Henceforth, it can be postulated that the level of inflammatory response is a major determinant of biocompatibility, and that addressing inflammation might reduce fibrosis around foreign body materials.

To date, acute inflammatory response following mesh implantation has often been evaluated in vitro and in the animal models through histologic and immunohistochemical studies [3–5]. In order to evaluate the precocious inflammatory response to mesh in vivo in humans, we chose to use a suitable model constituted by patients affected by a large median incisional hernia who underwent prosthetic hernia repair, using a sublay technique. This operation consists in a large dissection of both abdominal rectal muscles and their posterior sheath, in order to place the mesh, as well as a further dissection between subcutaneous tissue and sheath of the external oblique muscle [6].

The aim of this study was to evaluate, in the same patient, the acute inflammatory response in drain fluid produced near the prosthesis (DM) and in the subcutaneous space (DS) after prosthetic incisional hernia repair. For this reason, we evaluated some inflammatory indicators like cytokines (interleukins)[IL]-1, -10 and -1ra, C-Reactive Protein (CRP), total proteins, albumin and pH.

## Methods

The cohort of twelve male patients affected by midline incisional hernia with multiple hernial holes or with hernial defect of about 10 cm in size who, were admitted for surgical repair, were included in this study. We excluded patients ages >70 years, with co-morbidities like diabetes, hepatic cirrhosis, renal or heart failure, with infection or a potentially contaminated surgical field, and patients with necessity of a concomitant abdominal surgery.

All patients received an intravenous dose of 2 gr ceftadizime preoperatively that was administered two times daily for three days following surgery and low-molecular-weight heparin prophylaxis.

### Ethics

All participants gave a written informed consent. The ethics committee “Comitato etico dell’Azienda Ospedaliera Universitaria Policlinico Paolo Giaccone dell’Università degli Studi di Palermo” granted the exemption of the study from requiring ethics approval.

The research has been carried out adhering to the STROBE guidelines for observational studies.

### Surgical procedure

The operation starts with excision of the skin scar. The skin was separated from the surrounding tissue and wide undermining of the subcutaneous was done up to semilunar line. Excess sac was preserved. Medial edge of each rectus muscle was identified through palpation, and extreme medial edge of each rectus sheath was incised along its length to enter into the sub-muscular space.

This relatively bloodless plane could be developed easily to the lateral edges of the rectal muscle on each side. Dissection was stopped when an overlap of 5-6 cm to both lateral sides is reached. The vascular-nervous bundle of rectal muscle was identified to avoid damaging it during the preparation.

The posterior sheath of rectal muscle was secured with continuous sutures. A high density polypropylene mesh was placed between posterior sheath of rectal muscle and rectal muscle itself. The size of the mesh was 20 × 20 cm. Mesh was secured with 3-0 polypropylene stitches placed at the limits of dissection. A closed suction drain was placed over the mesh. In all cases both rectal muscles were closed to the median line and the anterior sheath was secured with separated stitches. Relaxing incision of the anterior rectal sheath were performed in all cases. Another closed suction drain was placed between the subcutaneous fat and oblique external sheath.

The wound drains were removed when the amount of drained fluid was less than 50 ml. The removal occurred in post-operative day (POD)-4 in 7 cases, in POD-5 in 3 cases and in POD-7 in other two cases. Drainage fluids were cultured only if infection was suspected (notably wound edema, erythema, or serosanguinous discharge), although this was not required in any patient included in the study. Body temperature was monitored by means of axillary temperature measurement 6 hours after surgery and on POD-1, 2 and 7.

Postoperative pain was assessed by the patient on mobilization by means of a visual analogue scale ranging from 0 (no pain) to 10 (worst pain imaginable) at 6, 24, 48, and 168 hours. Patients discharged before these times were requested to record pain at home. An analgesic therapy (oral tramadol hydrochloride) was administered on demand.

### Wound fluid samples and assays

Surgical wound fluids were assembled in closed sterile collection bags, which were replaced daily with new sterile bags under aseptic conditions. Wound fluids were collected on POD-1 until to POD-4. The amount of fluids collected for each patient was carefully recorded each time.

A sample of liquid was sent to the central laboratory of our department to determine pH, total proteins, albumin and CRP. A sample of this liquid were centrifuged at 2000 r for 10 minutes and stored at -70°C. Commercial enzyme-linked immunoadsorbant assay (ELISA) kits were used to determine the production of IL-10, -1 and -1ra (Euroclone, Wetherby, United Kingdom). The main test was a sandwich enzyme immune assay that uses a monoclonal antibody immobilized on a solid phase to capture antigen from the test specimen; peroxidase-conjugated monoclonal antibody was added to bind the antigens captured by the first antibody. At the time of the dosage, wound fluid samples were diluted where needed, and the assay protocol was performed by ELISA. The optical density for each well was determined using a microplate reader set at 450 nm with correction wavelength set at 540 nm.

Cytokines, total proteins, albumin and CRP quantity produced over 24 hours values were obtained multiplying their concentration by the volume collected in 24 hours. This procedure proved to be an accurate method in evaluating cytokine secretion because their production rate per milliliter varies according either the different method used or the pathology studied. Furthermore, cytokine secretion may change over time showing different values at the beginning and at the end of the observation time.

### Statistical analysis

All statistical analysis were performed using a statistical program (Graph Pad Instat Version 3.06 for Windows). Continous variable were given as mean and standard deviation. Unpaired t test with Welch correction was used to compare data from the two groups of variables. The variance analysis was performed by ANOVA with Bonferroni correction.

## Results

Age, ASA grade, diameter of hernial orifice, duration of operation, body mass index, and duration of hospital stay are shown in Table 1. All patients had uncomplicated intraoperative and postoperative course.

**Table 1:** **Demographic and clinical features of patients**

**Gender** (male)	n
	12
**Age** (years)	
Mean ± SD	44.2 ± 12.1
**ASA score**	n
I-II	9
III-IV	4
**BMI** (Mean ± SD)	28 ± 9.2
**Mean diameter of hernial orifice** (cm)	8.3 ± 1.9
**Mean duration of operation** (min)	92 ± 10.6
**Mean hospital stay** (days)	4.2 ± 0.9

Postoperative pain score values and analgesic consumption were higher in POD-1 and decreased in the following days. Body temperature peaking at 24 hours and returning to almost the same preoperative levels at 48 hours and no correlation was shown between temperature and markers of the inflammatory response (data not shown).

Clinical-instrumental controls up to a month after surgery showed no formation of seroma.

### Drain fluid

**Figure 1:**
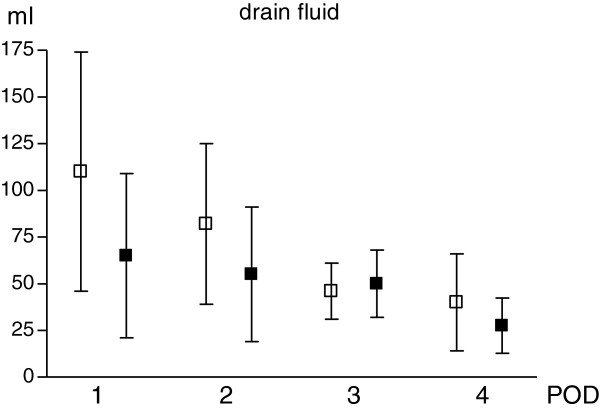
**Drain fluid collected after surgery in the two drains.** White square: DS; black square: DM Values are expressed s mean ± standard deviation. Unpaired t test with Welch correction was used. There are no significant differences among the two group for each assessment. The amount of drain fluid decreased significantly from POD-1 to POD-4 in both groups (p <0.01 in DS; p <0.05 in DM).

The characteristics of the fluid collected in the two drainage pouches was shown in Figure 1. In both drainage pouches, fluid was more copious on POD-1 and decreased progressively afterward. Initially the fluid had bloody characteristics, becoming serum like after. The pattern of drain fluid production was similar in both drains decreasing significantly from the POD-1 to POD-4, although the amount of fluid was higher in DS at all times. However, these higher rates of secretion were not statistically significant at various time.

### CRP, total protein, albumin and pH

**Figure 2:**
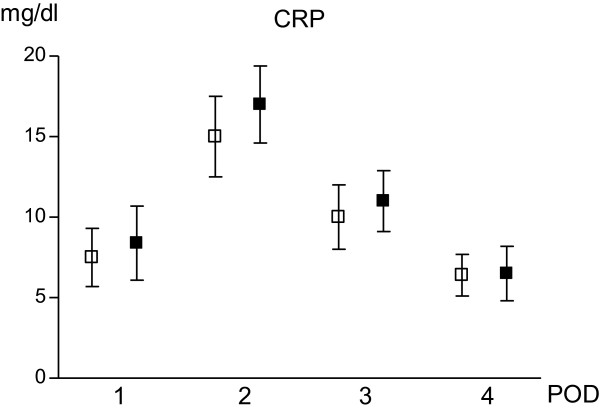
**CRP modifications after surgery in two drains.** White square: DS; black square: DM Values are expressed as mean ± standard deviation. Unpaired t test with Welch correction was used. There are no significant differences among the two group for each assessment. Significance in DS group: POD-1 vs POD-2 p <0.0001; POD-1 vs POD-3 p <0.01; POD-1 vs POD-4 p = n.s. Significance in DM group: POD-1 vs POD-2 p <0.0001; POD-1 vs POD-3 p <0.01; POD-1 vs POD-4 p = n.s.

The production of CRP showed a similar pattern in the fluid of both drains. CRP values resulted significantly increased in POD-2 if compared with POD-1 and reduced in POD-4. When comparing the two groups simultaneously, no significant differences were detected (Figure 2).

The amount of total protein and albumin resulted similar at all times and in comparison between DS and DM (data not shown).

**Figure 3:**
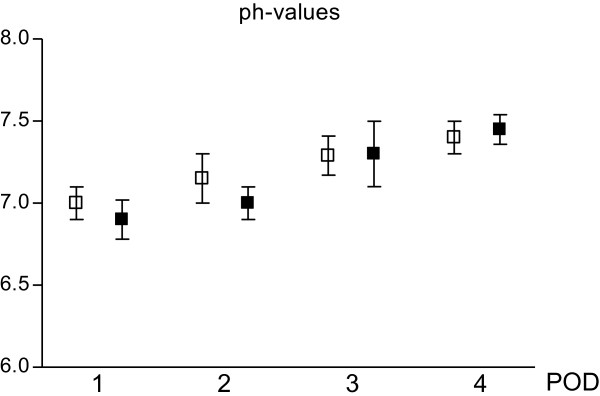
**ph-values tested on wound drainage fluid collected in 24 hours after surgery.** White square: DS; black square: DM. Values are expressed as mean ± standard deviation. Unpaired t test with Welch correction was used. There are no significant differences among the two group for each assessment. Significance in DM group: POD-1 VS POD-4 p <0.05.

PH values have not shown significant modifications in the DS. In the DM fluid pH values resulted significantly reduced in POD-4 when compared to those detected in POD-1 (p <0.05) (Figure 3).

### Cytokines in drain fluid

**Figure 4:**
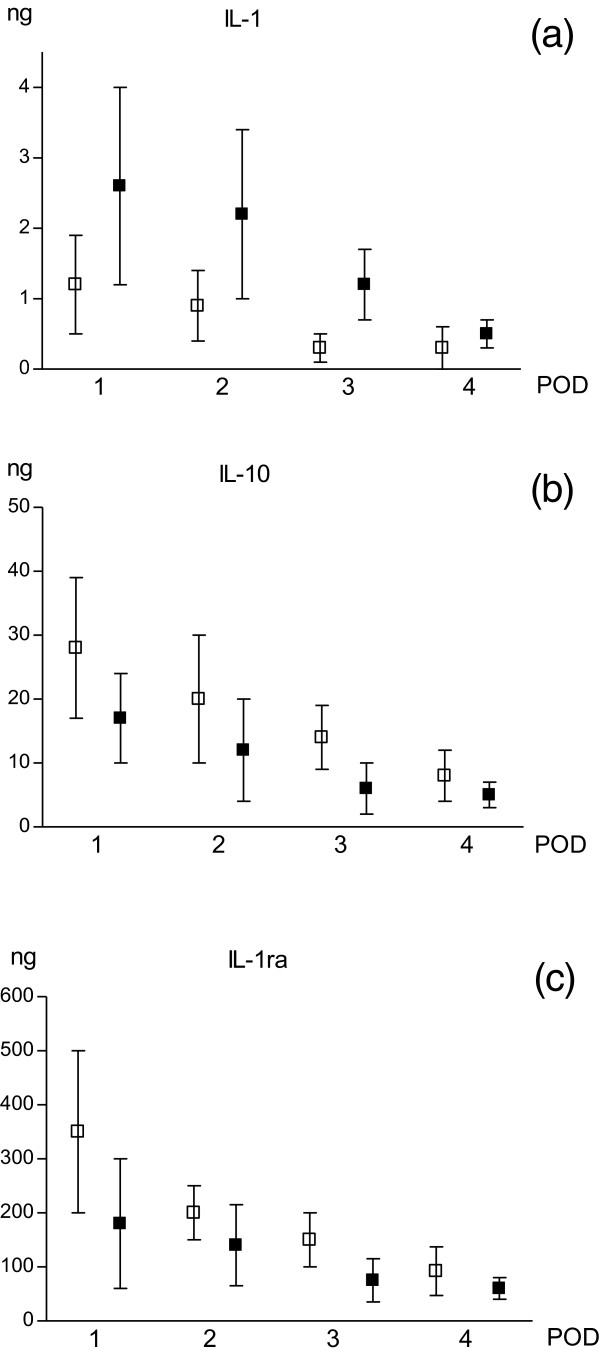
**Cytokines in drain fluid.** White square: DS; black square: DM. Values are expressed as mean ± standard deviation. Unpaired t test with Welch correction was used. DS vs DM for IL-1 **(a)**: POD-1 p <0.0001; POD-2 p <0.001; POD-3 p <0.01; POD-4 p <0.05. DS vs DM for IL-10 **(b)**: POD-1 p <0.01; POD-2 p <0.05; POD-3 p <0.001; POD-4 p = n.s. DS vs DM for IL-1ra **(c)**: POD-1 p <0.01; POD-2 p <0.05; POD-3 p <0.001; POD-4 p <0.05.

The dynamics of IL production resulted similar in both drainages (Figure 4).

In DS, no significant differences of IL-1 values were detected for all PODs. In DM, on the other hand, a significant reduction of IL-1 production value was reached in POD-3. Comparing DM with DS at all times, IL-1 values resulted significantly higher in DM (Figure 4a).

IL-1ra and IL-10 values in DM were similar in all PODs, whereas they resulted higher in POD-1 decreasing significantly starting from POD-2 in DS. Comparing DS and DM, both cytokines were always significantly higher in DS in POD-1, POD-2 and POD-3 (Figure 4b and c).

## Discussion

Wound healing is a dynamic process that involves a coordinated umoral response of many cell types representing distinct tissue compartments and is fundamentally similar among tissue types. The inflammatory phase of wound healing is characterized by the infiltration of neutrophils, macrophages, and lymphocytes, which participate by releasing pro-inflammatory and anti-inflammatory cytokines, growth factor, ingesting foreign materials, increasing vascular permeability, and promoting fibroblast activity [7, 8]. The presence of a foreign body as the mesh may alter the wound healing process [4]. Host reactions following implantation of biomaterials starts with blood/materials interactions and with provisional matrix formation, constituted by plasma proteins [9]. The progression of events is characterized by the extravasation and migration of monocytes/macrophages at the implant site with the release of cytokines, chemokines, growth and angiogenic factors etc.

Biomaterials immediately and spontaneously acquire a layer of host proteins prior to interacting with host cells. Thus it is highly probable that the types, levels, and surface conformations of the adsorbed proteins are critical determinants of the tissue reaction to such implants [10]. Conversely, the types, concentrations, and conformations of these surface-adsorbed proteins are dependent on biomaterial surface properties that dictate adhesion and survival of cells, especially monocytes and macrophages, on protein-coated surfaces [11].

The interaction of adsorbed proteins with adhesion receptors present on inflammatory cell populations, constitutes the major cellular recognition system for implantable synthetic materials. The presence of adsorbed proteins such as albumin, fibrinogen, complement, fibronectin, vitronectin, γ globulin and others, modulate host inflammatory cell interactions and adhesion and thus are linked to subsequent inflammatory and wound healing responses [12, 13].

The acute inflammatory response with biomaterials usually resolves quickly, in less than one week, depending on the extent of injury at the implant site. If present high levels and especially for prolonged periods of time may have a detrimental effect on healing [14].

The evaluation of some inflammatory response markers, in the drained fluid, allows us to better assess the events that follow a surgical wound [15]. However, the presence of a drain can itself modify the profiles of cytokines and wound healing. On the other hand, the levels of cytokines secreated near the surgical wound can be determined only by using the drainage, because the study of drain fluid is still the best clinical surrogate marker of healing [15]. Knowing the dynamic and the secretion profiles of different factors involved in the wound healing process at the site of surgical operation seems to be important because these soluble mediators play a crucial role in the pathophysiology of wound healing, as well as being popular target in the modification of the repair response [16].

This study shows that the inflammatory reaction differs in the subcutaneous and in the near-prosthetic space. High levels of anti-inflammatory interleukins (IL-10 and IL-1ra), and low levels of pro-inflammatory interleukin (IL-1) and CRP were detected in the DS fluid. Contrarily, in the DM fluid a low levels of anti-inflammatory interleukins and high levels of pro-inflammatory interleukin (IL-1) were observed.

In the DM fluid the elevated levels of pro-inflammatory cytokines promoted both a systemic and local inflammatory response directly or indirectly, causing fibro-proliferative lesions and maintaining inflammatory reaction and tissue scaring [17]. On the contrary, the higher IL-1ra and IL-10 production in DS fluid indicates that the inflammatory response is attenuated by the inhibitory activity of these anti-inflammatory cytokines [18–20]. The early production of IL-1, followed by its decrease by post-operative day, is in line with the essential role of this cytokine in the healing process because it increases collagen synthesis and stimulates keratinocytes and fibroblast growth [21].

Some studies indicate that a more intense acute inflammatory response determines the formation of strong scar tissue. Marois et al. [22] observed that a more intense inflammatory reaction early after implantation, stimulates significantly greater tissue in-growth and integration. Junge et al. [23] observed in experimental study on animal model after implantation of mesh, that the lesser degree of reaction was followed by less connective tissue formation and a higher partial volume of fat tissue.

It has been commonly observed in clinical practice of the patients, who previously underwent prosthetic incisional hernia repair with sublay technique, that the preparation of subcutaneous space results more easy and feasible with respect to the dissection of the space where there is the mesh, because in this case a strong scar tissue is represented.

The incidence of seroma after incisional hernia repair is high, reaching values of 3% [24]. The reasons for this are not known, however, high BMI, lowered preoperative serum concentration of total protein and albumin and high serum concentration of IL-1-RA are related to an elevated risk for postoperative seroma formation [24]. In this study, the small number of patients studied and the absence of seroma does not allow us to correlate the inflammatory response and the amount of fluid drained with the development of seroma. A significant reduction of pH values was detected in DM fluid only on POD-4. The pH value within the wound-milieu indirectly and directly influences all biochemical reactions which take place in the healing process. It could be proven that wound healing is correlated to wound pH changes, as they can lead to an inhibition of endogenous enzymes [25, 26], such as an inactivation of fibroblast bindering its wound healing activity [27]. For more than three decades the common assumption amongst physicians was that a low pH value, as found in normal skin, is favorable for wound healing. Recent investigation showed that the wound pH is indeed potent influential factor in the healing process [26]. A significant reduction in pH is associated with the formation of seroma [28] and the stabilization of the pH values can reduce the adverse tissue reaction [29].

## Conclusions

In conclusion, our data showed that an acute inflammatory reaction is present in both sites examined; however, in the site after mesh implantation it was significantly higher.

## Abbreviations

DM: Drain over Mesh
DS: Subcutaneous Drain
IL: Interleukin
CRP: C-Reactive Protein
POD: Post-Operative Day.
